# Antiamnesic Activity of an Ayurvedic Formulation Chyawanprash in Mice

**DOI:** 10.1093/ecam/neq021

**Published:** 2011-06-05

**Authors:** Milind Parle, Nitin Bansal

**Affiliations:** ^1^Pharmacology Division, Department of Pharmaceutical Sciences, Guru Jambheshwar University of Science & Technology, Hisar 125001, India; ^2^Rajendra Institute of Technology & Sciences, 4th milestone Hisar Road, Sirsa 125055, India

## Abstract

Chyawanprash (Chy) is an ayurvedic formulation commonly consumed in Indian households. Chy is a comprehensive herbal tonic, prepared from around 50 herbs employing anwala (*Emblica officinalis*) as the basic ingredient. The present study was undertaken to explore the beneficial effects of Chy (at the dose of 1 and 2% w/w of diet) administered daily for 15 successive days in mice with memory deficits. A total of 228 mice divided in 38 groups were employed in this study. Morris water maze, Hebb-Williams maze and elevated plus maze served as exteroceptive memory models, whereas scopolamine (Sco)-induced amnesia and alprazolam (Alp)-induced amnesia served as interoceptive memory models. The brain acetylcholinesterase activity, brain thiobarbituric acid reactive substances (TBARS) and reduced glutathione levels (GSH) were also estimated. The administration of Chy for 15 consecutive days significantly protected the animals from developing memory impairment. Furthermore, there was a significant decrease in brain TBARS and increase in GSH levels after administration of Chy (2% w/w), thereby indicating decreased free radical generation and increased scavenging of free radical, respectively. Thus, Chy may prove to be a useful remedy for the management of Alzheimer's disease owing to its antioxidant effect, pro-cholinergic action and/or antiamnesic potential.

## 1. Introduction

Learning and memory are two fundamental cognitive functions that confer us the ability to accumulate knowledge from our experiences [1]. Learning refers to the acquisition of any new information about the events occurring in surroundings and subsequent retrieval of this information is referred to as memory [2]. Memory is one of the most complex functions of the brain and ultimately involves multiple neuronal pathways and neurotransmitters. Impairment of memory is an organic brain disorder defined as “loss of intellectual ability of sufficient severity to interfere either with occupational functioning, usual social activities or relationship of a person in the absence of gross clouding of consciousness or motor involvement” [3]. Decreased cholinergic firing in brain [4], rise in oxidative stress [5], hypercholesterolemia [6], neuroinflammatory reactions [7] have been demonstrated to play an etiological role in memory decline. Since the allopathic system of medicine has yet to provide a radical cure for memory impairment, it is worthwhile to look for new directions, which would minimize the memory loss of patients with neuropsychiatric disorders.

Chyawanprash (Chy), a household remedy all over India, is popular for its nutritional value. In ayurveda, Chy is classified under the category of Rasayana, which aims at maintaining physique, vigor and vitality, while delaying the ageing process [8, 9]. It has been relished in India as a health food since ancient times with the same enthusiasm for the past 4000 years. Chy had been one of the most respected anti-ageing ayurvedic tonic, long before the clinical importance of vitamins, minerals and antioxidants was appreciated [8].

Chy is a complex mixture of more than 50 herbal ingredients. All the ingredients in Chy have been scientifically studied individually for their health benefits. The combination of these nutrients used in Chy in a specific quantity and manner of blending creates a powerful synergy for optimum health benefits. Chy is helpful in clearing the accumulated excreta by promoting digestion and excretion [8, 10]. It is not only hepatoprotective but it also streamlines the metabolism of fats and proteins [11, 12]. It relieves cough, asthma, bronchospasm, respiratory tract infections and tuberculosis [13]. It possesses promising antioxidant, cardiotonic, cholesterol lowering and anti-inflammatory properties [8, 10, 14, 15]. Chy is made in anwala base (Indian gooseberry, *Emblica officinalis*) [16], which is one of the richest sources of vitamin C (ascorbic acid) [17]. Anwala as well as ascorbic acid has been shown to be effective as memory enhancers in our earlier studies [18–20]. On similar lines, all formulations in which anwala or ascorbic acid are used as a base (or principal constituent) would produce beneficial effects on memory performance by virtue of their ascorbic acid content. Furthermore, no scientific data are available on the usefulness of Chy in the management of dementia till date to the best of our knowledge. In the light of above, the present study was undertaken to explore the antiamnesic potential of Chy.

## 2. Methods

### 2.1. Chy

Chy manufactured by Dabur, India was used in the present study. The proportion of various constituents of Chy is given in Table 1. Chy was administered orally for 15 successive days in two different doses (1 and 2% w/w) by admixing with the standard diet. The doses of Chy were determined on the basis of a pilot study and literature reports [8, 10, 14]. The animals were given the food *ad libitum*. The diet intake was measured daily by weighing the remaining diet (uneaten) in the cages and subtracting this amount from the total feed amount given on the previous day.

### 2.2. Drugs

The drugs and chemicals employed in this study were obtained from following drug houses: Chy (Dabur, India), donepezil hydrochloride (Donep; Wokhardt Ltd., Baddi, India), alprazolam (Alp) (Ind-Swift, Baddi, India), Sco hydrobromide (Sigma Aldrich, USA), 1,1,3,3-tertramethoxy propane (Sigma Aldrich, USA), 5,5-dithiobis-2-nitrobenzoic acid (DTNB) and reduced glutathione (Sisco Research Laboratories Pvt. Ltd., Mumbai, India), Piracetam (Nootropil, UCB India Ltd, India), acetylcholine iodide, eserine salicylate, sodium dihydrogen phosphate, disodium hydrogen phosphate (Hi-Media, India). Sco HBr and Donep HCl were dissolved in distilled water. Alp was suspended in 1% carboxymetylcellulose sodium.

### 2.3. Animals

Swiss male mice (20–25 g, 3-4 months of age) were employed in this study, as consequent variation in estrogen levels in female mice may influence the cognitive behavior of the animals [21]. All the animals were procured from the disease-free small animal house of CCS Haryana Agriculture University, Hisar, Haryana, India. The animals had free access to food and water. They were subjected to a natural light-dark (12 h each) cycle. The animals were acclimatized for at least 5 days to the laboratory conditions prior to behavioral experiments. Experiments were carried out between 09:00 and 18:00 h. The experimental protocol was approved by the Institutional Animal Ethics Committee and the care of the laboratory animals was taken as per the guidelines of CPCSEA, Ministry of Forests and Environment, Government of India.

### 2.4. Laboratory Models for Testing Memory

1. Exteroceptive behavioral models, such as (i) Morris water maze (MWM), (ii) Hebb-Williams maze (HWM) and (iii) elevated plus maze (EPM), and
2. Interoceptive behavioral models, such as (i) Sco-induced amnesia (1.4 mg kg^−1^; i.p.) [22] and (ii) Alp-induced amnesia (0.5 mg kg^−1^; i.p.) were employed in the present study [23, 24].

### 2.5. MWM

The procedure, technique and end point for testing memory were followed as per the parameters described earlier [23, 24]. Briefly, MWM consisted of a circular pool (150 cm in diameter, 45 cm in height) filled to a depth of 30 cm with water maintained at 25°C. The water was made opaque with non-toxic white colored dye. The tank was divided into four equal quadrants with the help of two threads, fixed at right angle to each other on the rim of the pool. A submerged platform (10 cm^2^), painted in white was placed inside the target quadrants (Q4 in present study) of this pool 1 cm below surface of water. The position of platform was kept unaltered throughout the training session. Each animal was subjected to four consecutive trials each day with a gap of 5 min for four consecutive days (starting from 15th day of Chy administration to 18th day), during which they were allowed to escape on to the hidden platform and to remain there for 20 s. If the mouse failed to find the platform within 120 s, it was guided gently onto the platform and allowed to remain there for 20 s. Escape latency time (ELT) was defined as the time taken by the animal to locate the hidden platform. ELT was noted as an index of learning.

On fifth day (i.e., 19th day of Chy administration), the platform was removed. Mouse was placed in water maze and allowed to explore the maze for 120 s. Each mouse was subjected to four such trials and each trial was started from a different quadrant. Mean time spent in all the three quadrants, that is, Q1, Q2 and Q3 was recorded and the time spent in the target quadrant (TSTQ) in search of the missing platform provided as an index of retrieval. Care was taken not to disturb the relative location of water maze with respect to other objects in the laboratory.

### 2.6. HWM

HWM is an incentive-based exteroceptive behavioral model useful for measuring spatial working memory of rodents. The procedure, technique and end point for testing memory were followed as per the parameters described earlier in our studies [23, 25]. Briefly, HWM consists of mainly three components, (i) animal chamber (or start box), which is attached to (ii) the middle chamber (or exploratory area) and (iii) a reward chamber at the other end of the maze in which reward (food) is kept. All the three components were provided with guillotine removable doors. On the first day (i.e., 15th day of Chy administration), the mouse was placed in the animal chamber and the door was opened to facilitate the entry of animal into the next chamber. The door of the start box was closed immediately after the animal moved into the next chamber so as to prevent back entry. Time taken by the animal to reach reward chamber (TRC) from start box on 1st day reflected the learning index. Each animal was allowed to explore the maze for 3 min with all the doors opened before returning to home cage. Retention (memory score) of this learned task was examined 24 h after the first-day trial. Significant reduction in TRC value indicated improvement of memory.

### 2.7. EPM

EPM served as the exteroceptive behavioral model to evaluate short-term memory in mice. The procedure, technique and end point for testing memory was followed as per the parameters described earlier [23, 26]. Briefly, EPM for mice consisted of two open arms (16 cm × 5 cm) and two covered arms (16 cm × 5 cm × 12 cm) extended from a central platform (5 cm × 5 cm), and the maze was elevated to a height of 25 cm from the floor. On the first day (i.e., 15th day of Chy administration), each mouse was placed at the end of an open arm, facing away from the central platform. Transfer latency (TL) was defined as the time (in seconds) taken by the animal to move from the open arm into one of the covered arms with all its four legs. The mouse was allowed to explore the maze for another 2 min and then returned to its home cage. Retention of this learned task (memory) was examined 24 h after the learning trial. Significant reduction in TL value indicated improvement of memory.

### 2.8. Measurement of Locomotor Activity

The effect of Chy on ambulation (spontaneous locomotor activity) was recorded using Medicraft photoactometer (INCO, Ambala, India) in different groups of mice.

### 2.9. Experimental Design

Mice were divided in 38 groups comprising of six animals in each group. Chy was administered for 15 days. After completion of 15 days, behavioral studies were carried out using MWM (Groups I–XII), HWM (Groups XIII–XXIV) and EPM (Groups XXV–XXXVI). Chy administration was continued during maze studies, that is, Chy was administered for 16 days in case of EPM and HWM and for 19 days in case of MWM. The animals had free access to food and water before subjecting them to behavioral studies using mazes. Piracetam is an established memory enhancer [14, 27] available in market, it served as positive control to compare the memory enhancing activity of Chy in all the exteroceptive behavioral models.

Groups I, XIII, and XXV (control groups): Standard diet (without Chy) was administered to mice.

Groups II and III, XIV and XV and XXVI and XXVII (Chy groups): Chy in two concentrations (1 and 2% w/w in the diet) was administered to mice daily for 15 successive days.

Groups IV, XVI, and XXVIII (piracetam groups): Piracetam (400 mg kg^−1^; i.p.) was administered for 15 successive days to mice.

Groups V, XVII, and XXIX (Sco group): Sco (1.4 mg kg^−1^; i.p.) was administered daily from (days 1 to 4) 30 min before training on water maze.

Groups VI and VII, XVIII and XIX and XXX and XXXI (Chy + Sco groups): Chy in two concentrations (1 and 2% w/w in the diet) was administered to mice daily for 15 successive days. Sco (1.4 mg kg^−1^; i.p.) was injected after 90 min of Chy administration daily from days 15 to 18.

Groups VIII, XX, and XXXII (Piracetam + Sco group): Piracetam (400 mg kg^−1^; i.p.) was administered for 15 successive days to mice. Sco (1.4 mg kg^−1^; i.p.) was injected after 90 min of piracetam administration daily from days 15 to 18.

Groups IX, XXI and XXXIII (Alp group): Alp (0.5 mg kg^−1^; i.p.) was administered daily from (days 1 to 4) 30 min before training on water maze.

Groups X and XI, XXII and XXIII and XXXIV and XXXV (Chy + Alp groups): Chy in two concentrations (1 and 2% w/w in the diet) was administered to mice daily for 15 successive days. Alp (0.5 mg kg^−1^; i.p.) was injected after 90 min of Chy administration daily from days 15 to 18.

Groups XII, XXIV and XXXVI (Piracetam + Alp group): Piracetam (400 mg kg^−1^; i.p.) was administered for 15 successive days to mice. Alprazoalm (0.5 mg kg^−1^; i.p.) was injected after 90 min of piracetam administration daily from days 15 to 18.

After behavioral studies, animals were sacrificed by decapitation. The whole brain was removed, weighed and homogenized in an ice bath after adding 10 volumes of phosphate buffer (pH 7.4). The homogenate was centrifuged at 3000 rpm for 15 min and the resultant cloudy supernatant fluid was used for estimation of acetylcholinesterase (AChE), reduced glutathione (GSH) and thiobarbituric acid reactive substances (TBARS). Since Donep is a marketed preparation for the management of Alzheimer's disease (AD), which acts through inhibition of acetyl-cholinesterase enzyme [3, 28], it served as a standard drug for confirming AChE inhibitory activity of Chy. Furthermore, Donep has also been reported to possess antioxidant activity [3, 28], it served as a standard drug for comparing TBARS and GSH levels.

Group XXXVII (Donep group): Donep (0.1 mg kg^−1^; i.p.) was administered for 15 days daily to mice. Estimation of brain AChE, GSH and TBARS was carried out on 15th day after 90 min of Donep administration.

Group XXXVIII (Donep + Sco group): Donep (0.1 mg kg^−1^; i.p.) was administered for 15 days daily to mice. Sco (1.4 mg kg^−1^; i.p.) was injected on 15th day after 90 min of Donep administration. Estimation of brain AChE, GSH and TBARS was carried out on 15th day after 45 min of Sco administration.

### 2.10. Estimation of Brain AChE Levels

The whole brain AChE activity was measured by the method of Ellman et al. with a slight modification [29, 30]. The change in absorbance per minute of the sample was recorded spectrophotometrically at 420 nm.

### 2.11. Estimation of Brain GSH Levels

The whole brain GSH level was measured by the method of Beutler et al. [31]. The absorbance was measured spectrophotometrically at 412 nm.

### 2.12. Estimation of Brain TBARS Levels

The whole brain TBARS level was measured by the method of Ohkawa et al. [32]. The absorbance was measured spectrophotometrically at 532 nm.

### 2.13. Statistical Analysis

All the results were expressed as mean ± SEM. Data were analyzed using one way analysis of variance followed by *post hoc* Tukey's multiple range test. *P* < .05 was considered to be statistically significant.

## 3. Results

The administration of Chy has not affected the diet intake and weight of the animals in comparison to control group animals in the present study.

### 3.1. Chy Prevented Development of Memory Deficits

**Figure 1:**
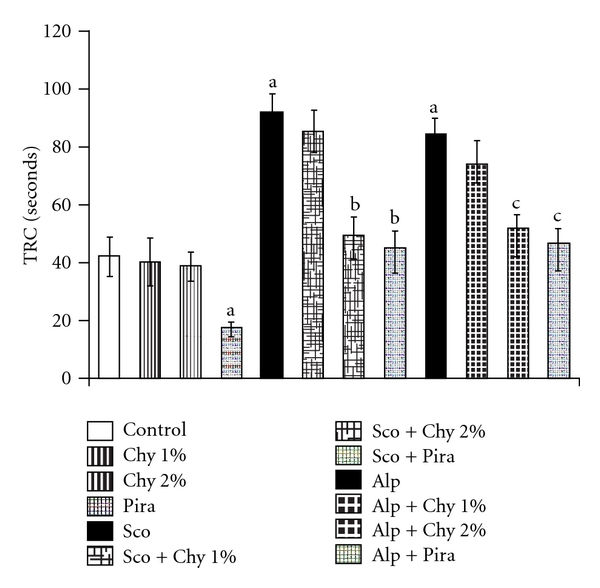
Effect of Chy administered for 15 successive days on time taken by mice to reach reward chamber (TRC) using HWM. Piracetam (Pira) (400 mg kg^−1^; i.p.) is used as a standard drug. Values are mean ± SEM; ^a^ *P* < .05 as compared to control group; ^b^ *P* < .05 as compared with Sco group; ^c^ *P* < .05 as compared with Alp group.

**Figure 2:**
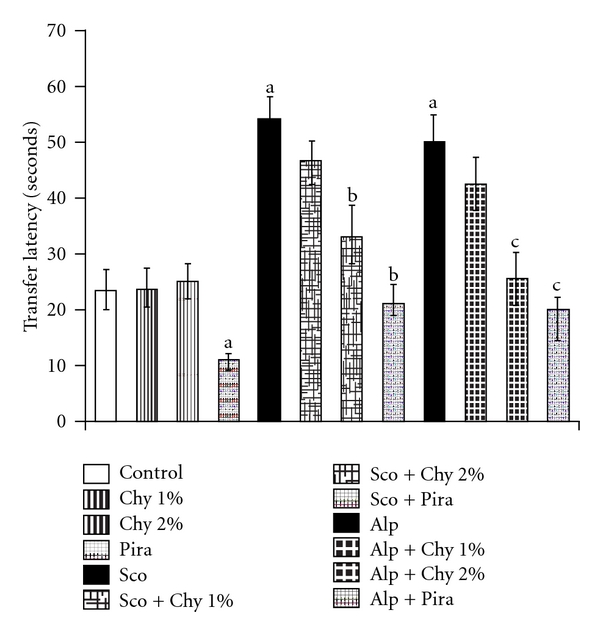
Effect of Chy administered for 15 successive days on TL of mice using EPM. Piracetam (Pira) (400 mg kg^−1^; i.p.) is used as a standard drug. Values are mean ± SEM; ^a^ *P* < .05 as compared to control group; ^b^ *P* < .05 as compared with Sco group; ^c^ *P* < .05 as compared with Alp group.

The administration of Sco (1.4 mg kg^−1^; i.p.) and Alp (0.5 mg kg^−1^; i.p.) before training trials induced amnesia in mice using MWM (Table 2). Furthermore, Sco and Alp administration to mice significantly increased both TRC (Figure 1) and TL (Figure 2) when recorded using HWM and EPM, respectively. These observations suggested that Sco and Alp had produced impairment in learning as well as memory. However, Chy alone did not produce any significant improvement in memory scores of mice in these behavioral models (Figures 1 and 2]. But, Sco and Alp induced memory deficits were successfully reversed by Chy (1 and 2% w/w) as indicated by decreased day 4 ELT, increased day 5 TSTQ (Table 2), decreased TRC (Figure 1) and decreased TL (Figure 2). Piracetam (400 mg kg^−1^; i.p.) served as the positive control and pretreatment with piracetam reversed Sco- and Alp-induced memory deficits as expected (*P* < .05).

### 3.2. Brain AChE Activity

**Figure 3:**
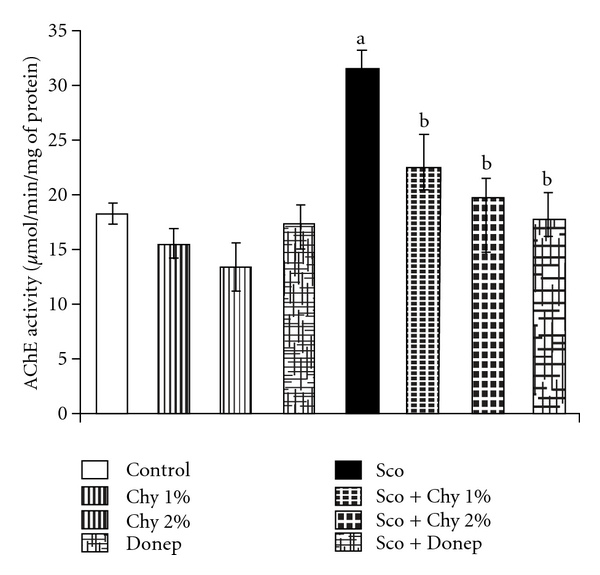
Effect of Chy administered for 15 successive days on brain AChE activity. Donep (0.1 mg kg^−1^; i.p.) is used as a standard drug. Values are mean ± SEM; ^a^ *P* < .05 as compared to control group; ^b^ *P* < .05 as compared with Sco group.

No statistically significant differences were observed in brain AChE activity of Chy-treated mice and control group mice. On the other hand, administration of Sco (1.4 mg kg^−1^, i.p.) significantly increased the brain AChE activity, which was reversed (*P* < .05) by Chy administered chronically for 15 days. Donep (0.1 mg kg^−1^; i.p.) used as a standard drug, showed decrease in brain AChE activity of mice as expected (Figure 3).

### 3.3. Antioxidant Studies

**Figure 4:**
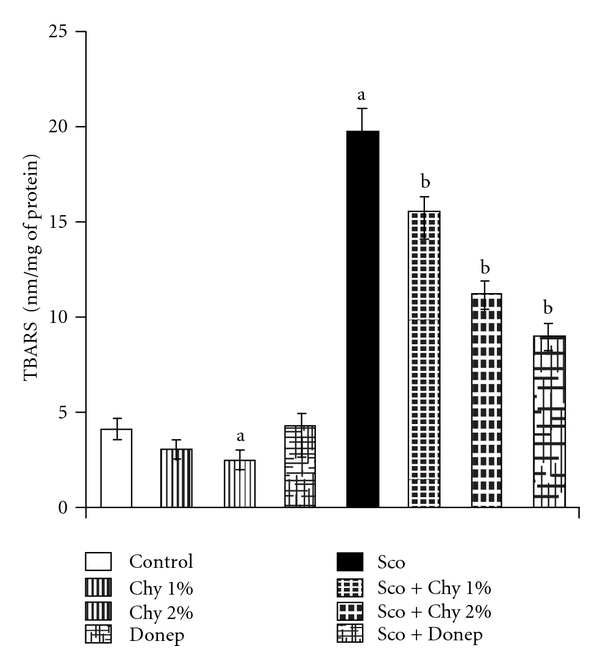
Effect of Chy administered for 15 successive days on brain TBARS. Donep (0.1 mg kg^−1^; i.p.) is used as a standard drug. Values are mean ± SEM; ^a^ *P* < .05 as compared to control group; ^b^ *P* < .05 as compared with Sco group.

**Figure 5:**
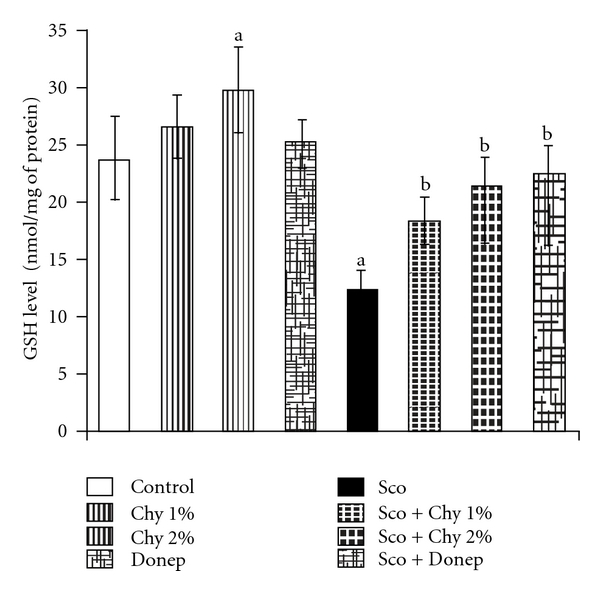
Effect of Chy administered for 15 successive days on brain GSH level. Donep (0.1 mg kg^−1^; i.p.) is used as a standard drug. Values are mean ± SEM; ^a^ *P* < .05 as compared to control group; ^b^ *P* < .05 as compared with Sco group.

No statistically significant differences were observed in TBARS levels (Figure 4) and GSH levels (Figure 5) in mice treated with Chy (1% w/w). But, Chy (2% w/w) significantly diminished (*P* < .05) brain TBARS levels and enhanced brain GSH levels as compared to the control group. Administration of Sco (1.4 mg kg^−1^; i.p.) not only increased the brain TBARS levels but decreased brain GSH levels, when compared to control group (*P* < .05). Administration of both Chy and Donep significantly reversed the Sco-induced increase in brain TBARS levels (Figure 4) and decrease in brain GSH levels (Figure 5).

**Figure 6:**
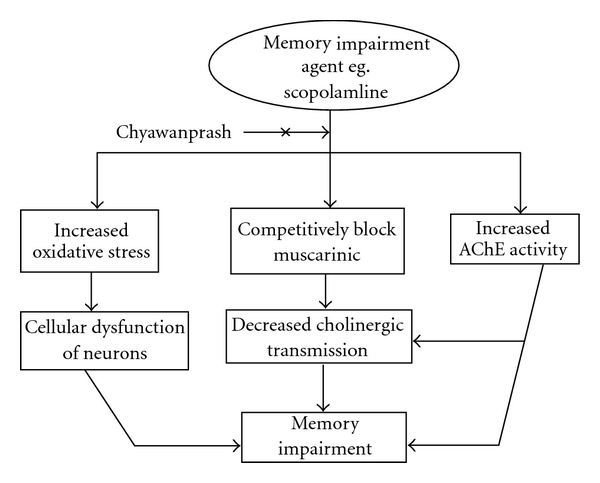
Schematic diagram illustrating the beneficial effect of Chy on memory.

In the present study, Alp did not affect significantly the brain AChE activity, TBARS levels and GSH levels in any manner. Chy (1 and 2% w/w in the diet) when administered for 15 successive days did not show any significant change in locomotor activity of mice (scores: 193 ± 12 and 202 ± 14) as compared to control animals (score: 178 ± 09). The schematic diagram illustrating the beneficial effect of Chy on memory is depicted in Figure 6.

## 4. Discussion

Dementia is a clinical syndrome characterized by the development of multiple cognitive defects that are severe enough to interfere with daily social and professional functioning [33]. AD is the most common cause of dementia in the elderly, accounting for 60–70% of all demented cases. AD-related dementias are neurodegenerative conditions characterized by progressive brain dysfunction occurring in a step-wise biologic sequence: neuronal injury; synaptic failure and neuronal death. Neurofibrillary tangles, amyloid plaques and degeneration of cholinergic neurons are the pathological hallmarks of AD [34]. To improve cholinergic transmission, different strategies are adopted, including increase of ACh synthesis, the augmentation of presynaptic ACh release, and the stimulation of cholinergic post synaptic muscarinic and nicotinic receptors and the inhibition of ACh synaptic degradation by employing cholinesterase inhibitors [35]. Despite the availability of various treatment strategies, the severity and prevalence of this disease are not yet under control. Therefore, alternative and complementary medicines including herbal supplements are being utilized in the management of this disease [18, 36].

In the present study, Sco and Alp produced amnesia in experimental animals as indicated by the increased day 4 ELT, reduced TSTQ (time spent to reach target quadrant) using MWM, increased TL using EPM and increased TRC (time taken to reach reward chamber) using HWM. Sco (centrally acting anti-muscarinic drug) and Alp (a benzodiazepine) have been extensively utilized to induce memory deficits in experimental animals [37, 38]. Alp and other benzodiazepines produce amnesia in laboratory animals by activation of benzodiazepine receptors [39, 40]. Pretreatment with Chy orally for 15 successive days prevented memory loss of mice as reflected by the decreased day 4 ELT, increased TSTQ using MWM, reduced TL using EPM and decreased TRC using HWM as compared to respective control animals. In the present study, it was observed that Chy failed to improve memory of normal animals. Alp-induced memory deficits were successfully reversed by Chy, when administered for 15 consecutive days. These findings suggest that Chy may be serving as a protective agent in some manner which was successful in preventing the damage elicited by Alp.

Sco-induced amnesia is mediated through blockade of central muscarinic receptors [24]. Since Chy reversed Sco-induced amnesia in the present study, it appears that Chy might have produced either competitive displacement of Sco from cholinergic (muscarinic) receptors or stimulated in some manner the synthesis of acetylcholine. The antiamnesic effect of Chy may be due to the presence of anwala (*E. officinalis*) in abundant quantity. This suggestion is confirmed by our earlier studies where an ayurvedic formulation comprising principally of Anwala Churna produced a dose-dependent improvement in memory scores and reduction in brain cholinesterase activity of young and aged mice [14, 20]. Anwala is the one of the richest source of Vitamin C [17]. Parle and Dhingra [19] had shown vitamin C (ascorbic acid) to be a promising memory enhancer. In this study, ascorbic acid administered for 8 consecutive days not only improved learning and memory in aged mice but also protected young mice from Sco and diazepam induced impairment of memory. On similar lines, all formulations in which anwala (or ascorbic acid) is used as a base (or principal constituent) would produce beneficial effects on memory performance by virtue of ascorbic acid content. This also explains the underlying mechanism of Chy which has reversed the memory deficits induced by Sco and Alp.

An increased lipid peroxidation due to increased generation of free radicals and decreased scavenging of free radicals due to reduced antioxidant enzymes has been reported in AD brain [41, 42]. Sco significantly increased the oxidative stress as indicated by the increased TBARS and decreased GSH levels in the present study. This observation is supported by several reports in literature [22, 43, 44]. The administration of Chy for 15 successive days to mice not only decreased oxidative stress but also prevented the Sco-induced rise in oxidative damage as indicated by the reduced TBARS and increased GSH levels as compared to respective control animals. *Emblica officinalis* [17], *Withania somnifera* [45], *Nelumbium speciosum* [46], *Sesamum indicum* [47], *Cinnamomum tamala* [48], *Vitis vinifera* [49], *Piper longum* [50], *Sida cordifolia* [51], *Terminalia chebula* [52], *Aegle marmelos*, *Ipomoea digitata*, *Phyllanthus niruri*, *Tinospora cordifolia* and *Boerhaavia diffusa* [53] are individually reported to possess antioxidant activity. Therefore, it seems likely that Chy may prove to be a useful antioxidant by virtue of its inhibitory effect on free radical generation and stimulatory effect on scavenging of free radicals. In the present study, Alp did not affect significantly the brain AChE, TBARS and GSH levels in any manner probably because the mechanism of action of Alp is related to benzodiazepine receptors and GABA activity.

It is noteworthy that phytoconstituents such as *E. officinalis* [17] and *W. somnifera* [45] present in Chy have been reported to be potent antioxidants. The phytoconstituents such as *Eugenia caryophyllus* [54] and *V. vinifera* [55] possess potent anticholinesterase activity (pro-cholinergic action), which may be responsible for the nootropic effect of Chy. In addition, *W. somnifera* [56], *T. cordifolia* [57], *E. officinalis* [20] and *P. longum* [58] are also reported to improve memory and memory deficits. In the light of above discussion, it may be concluded that Chy induced antiamnesic effect may be related to its pro-cholinergic and/or antioxidant activities. However, further investigations focused on potential effects of Chy on specific genes (by gene-expression analyses especially in the hippocampus) and other neurotransmitters involved in reversing memory defects are essential.

## Figures and Tables

**Table 1:** Proportion of constituents of Chy.

Common name	Plant name	Weight per 100 g of Chy
Dashmool	*Aegle marmelos* Corr.	3.98 g
	*Desmodium bulbifera* Desv.	
	*Gmelina arborea* Roxb.	
	*Oroxylum indicum* Vent.	
	*Premna integrifolia* Linn.	
	*Solanum indicum* Linn.	
	*Solanum xanthocarpum* Schard.	
	*Stereospermum suoveolens* DC	
	*Tribulus terrestris* Linn.	
	*Uraria picta* Desv.	
Bala	*Sida cordifolia* Linn	0.398 g
Mudgaparni	*Phaseolus tribolus-sensu* Ait	0.398 g
Mashparni	*Teramnus labialis* Spreng	0.398 g
Karkatshringi	*Pistacia integerima* Stew-ex Brandis	0.398 g
Bhumiamlaki	*Phyllanthus niruri* Linn.	0.398 g
Jivanti	*Leptadenia reticulata* Wight & Am	0.398 g
Pushkarmool	*Inula racemosa* Hook.	0.398 g
Agarkashta	*Aquillaria agallocha* Roxb.	0.398 g
Haritaki	*Terminalia chebula* Retz.	0.398 g
Guruchi	*Tinospora cordifolia* Meirs	0.398 g
Katchur	*Curcuma zedoaria* Rosc	0.398 g
Mustak	*Cyperus rotundus* Linn	0.398 g
Punarnava	*Boerhaavia diffusa* Linn	0.398 g
Neelkamal	*Nelumbium speciosum* Willd	0.398 g
Vasaka	*Adhatoda vasica* Nees.	0.398 g
Kakanasa	*Martynia diandra* Glox	0.398 g
Yashtimadhu	*Glycyrrhiza glabra* Linn	0.398 g
Varahikand	*Dioscorea bulbifera* Linn	0.796 g
Ashwagandha	*Withania somnifera* Dunal	0.796 g
Satavari	*Asparagus racemosus* Willd	0.796 g
Vidarikand	*Ipomoea digitata* Linn	1.195 g
Anwala green	*Emblica officinalis* Gaertn	90 g
Ghrit	Milk fat	2.08 g
Sesame oil	*Sesamum indicum* Linn	1.20 g
Banslochan	*Bambusa arundinacea* Willd	0.8 g
Akarkara	*Spilanthes acmella* Linn	0.126 g
Pippali	*Piper longum* Linn.	1.12 g
Nagkesar	*Mesua ferrea* Linn.	0.116 g
Dalchini	*Cinnamomum zeylanicum* Breyn	0.116 g
Tejpatra	*Cinnamomum tamala* Nees & Ebrm	0.116 g
Lavang	*Eugenia caryophyllus* Linn	0.128 g
Elaichi	*Elettaria cardamomum* Maton	0.588 g
Kesar	*Crocus sativus* Linn.	0.021 g
Chandansaar	*Ptertocarpus santalinus* Linn	0.0092 g
Abhrak Bhasam	Ayurvedic preparation	0.188 g
Muktasukti Pishti	Ayurvedic preparation	0.063 g
Silver foil	*—*	q.s.
Sugar	*—*	q.s.

q.s.: quantity sufficient.

**Table 2:** Effect of Chy on day 4 ELT and day 5 time spent in target quadrant (TSTQ) of mice using MWM.

Group	Treatment	Dose	Day 1 ELT (s)	Day 4 ELT (s)	Day 5 TSTQ (s)
I	Control (normal)	Standard diet	69.7 ± 2.4	25.6 ± 1.8^a^	73.4 ± 3.6
II	Chy	1% w/w, in diet	68.6 ± 1.6	24.2 ± 2.4	70.6 ± 4.2
III	Chy	2% w/w, in diet	65.6 ± 0.6	26.7 ± 2.9	72.8 ± 2.4
IV	Piracetam	400 mg kg^−1^; i.p.	67.9 ± 3.9	13.4 ± 3.1^b^	89.9 ± 2.2^e^
V	Sco	1.4 mg kg^−1^; i.p.	66.3 ± 3.5	56.2 ± 3.6^b^	29.2 ± 4.9^e^
VI	Chy + Sco	1% w/w, in diet + 1.4 mg kg^−1^; i.p.	64.3 ± 1.2	34.8 ± 2.1^c^	52.1 ± 1.8^f^
VII	Chy + Sco	2% w/w, in diet + 1.4 mg kg^−1^; i.p.	62.1 ± 4.3	25.4 ± 1.3^c^	61.2 ± 4.6^f^
VIII	Piracetam + Sco	400 mg kg^−1^; i.p. + 1.4 mg kg^−1^; i.p.	62.3 ± 2.9	14.4 ± 2.7^c^	66.3 ± 2.8^f^
IX	Alp	0.5 mg kg^−1^; i.p.	63.4 ± 2.9	59.9 ± 4.3^b^	24.3 ± 1.2^e^
X	Chy + Alp	1% w/w, in diet + 0.5 mg kg^−1^; i.p.	63.7 ± 2.7	32.1 ± 1.6^d^	54.2 ± 3.2^g^
XI	Chy + Alp	2% w/w, in diet + 0.5 mg kg^−1^; i.p.	60.1 ± 2.5	22.1 ± 4.1^d^	64.6 ± 3.7^g^
XII	Piracetam + Alp	400 mg kg^−1^; i.p. + 0.5 mg kg^−1^; i.p.	66.3 ± 1.9	17.3 ± 2.3^d^	69.1 ± 3.6^g^

Piracetam (400 mg kg^−1^; i.p.) is used as a standard drug. Values are mean ± SEM.

^a^ *P* < .05 as compared to day 1 ELT in control.

^b^ *P* < .05 as compared to day 4 ELT in control.

^c^ *P* < .05 as compared with day 4 ELT in Sco group.

^d^ *P* < .05 as compared with day 4 ELT in Alp group.

^e^ *P* < .05 as compared to day 5 TSTQ in control.

^f^ *P* < .05 as compared with day 5 TSTQ in Sco group.

^g^ *P* < .05 as compared with day 5 TSTQ in Alp group.
